# Enfortumab vedotin plus pembrolizumab as a first-line treatment for advanced urothelial carcinoma: a cost-effectiveness analysis from China based on the EV-302 trial

**DOI:** 10.3389/fphar.2024.1412292

**Published:** 2024-09-26

**Authors:** Maojin You, Qiaoyan Zheng, Ying He

**Affiliations:** ^1^ Department of Pharmacy, Mindong Hospital Affiliated to Fujian Medical University, Ningde, Fujian, China; ^2^ Department of Pathology, Mindong Hospital Affiliated to Fujian Medical University, Ningde, Fujian, China; ^3^ Department of Emergency Medicine, Mindong Hospital Affiliated to Fujian Medical University, Ningde, Fujian, China

**Keywords:** cost-effectiveness, enfortumab vedotin, pembrolizumab, urothelial carcinoma, first-line treatment

## Abstract

**Background:**

The efficacy and safety of enfortumab vedotin combined with pembrolizumab (EV-PEMB) was investigated as a first-line treatment for advanced urothelial carcinoma (UC) in a phase III clinical trial (EV-302). The trial findings indicated significant prolonged progression-free survival (PFS) and overall survival (OS) compared to chemotherapy with a favorable safety profile. However, EV-PEMB is costly and it is unknown whether it is cost-effective compared to chemotherapy. This study aimed to conduct a cost-effectiveness analysis of EV-PEMB *versus* chemotherapy as a first-line treatment for advanced UC from the perspective of the Chinese healthcare system.

**Methods:**

A Markov model with three distinct health states was developed to assess the cost-effectiveness of EV-PEMB as a first-line treatment for advanced UC *versus* chemotherapy based on the EV-302 trial. Drug costs were obtained from national tender prices. Other expenses and utility values were sourced from the literature or expert advice. The findings of the study included total costs, quality-adjusted life years (QALYs), and incremental cost-effectiveness ratios (ICERs). We conducted a one-way sensitivity analysis and probabilistic sensitivity analysis to ensure the model’s robustness.

**Results:**

The EV-PEMB regimen demonstrated a gain of 3.22 QALYs at $375,420.24, compared to the chemotherapy regimen with 1.70 QALYs at $23,369.67. ICER for EV-PEMB compared to chemotherapy was at $232,256.16 per QALY gained. In China, at a willingness-to-pay threshold of $38,133 per QALY, EV-PEMB has a 0% probability of being cost-effective as a first-line treatment for advanced UC compared to chemotherapy.

**Conclusion:**

From the perspective of the Chinese healthcare system, EV-PEMB is unlikely to be a cost-effective first-line treatment option for advanced UC compared to chemotherapy.

## 1 Introduction

Urothelial carcinoma (UC) is an epithelial cancer caused due to a malignant transformation of the overlying epithelium of the urinary tract (NCCN, 2022). UC has a non-negligible morbidity and mortality rate worldwide (Wong et al., 2018). UC can occur in any part of the urinary system, and more than 90% of the cases involve the bladder (Cathomas et al., 2022). In 2020, more than 573,000 new cases of bladder cancer were reported, with approximately 213,000 associated deaths, making it the 10th most malignant tumor globally (Sung et al., 2021). Early-stage UC is curable; however, more than 5% of patients with UC are first diagnosed with locally advanced or metastatic disease and typically show a poor prognosis (Lopez-Beltran et al., 2021), with 5-year survival rates of only 34% and 5.4%, respectively (National Cancer Institute, 2023). Platinum-based chemotherapy is the first-line treatment for advanced UC; however, treatment outcomes remain unsatisfactory (Powles et al., 2024). For instance, patients receiving cisplatin-based chemotherapy have a median progression-free survival (PFS) of only 7–9 months and a median overall survival (OS) of approximately 16–18 months (El Rassy et al., 2019; Sorce et al., 2022; van der Heijden et al., 2023; Powles et al., 2024). Immune checkpoint inhibitors (ICIs), such as nivolumab and pembrolizumab, also failed to provide significant clinical benefit in advanced UC (van der Heijden et al., 2023; Flaig et al., 2022; Powles et al., 2021). Therefore, there is a need for a new treatment option for patients with advanced UC.

Enfortumab vedotin (EV) is an antibody-drug conjugate (ADC) known to induce cell cycle arrest and cell death (O'Donnell et al., 2023). It confers survival advantages to patients with locally advanced or metastatic UC who have received prior therapy (Yu et al., 2021). Preclinical data suggest that EV, in combination with pembrolizumab, can augment antitumor activity synergistically, leveraging their respective mechanisms of action to achieve complementary efficacy (O'Donnell et al., 2023). Recently, in a phase III clinical trial (EV-302) (Powles et al., 2024) involving previously untreated patients with advanced UC, EV combined with pembrolizumab (EV-PEMB) demonstrated enhanced clinical benefits and a manageable safety profile compared to chemotherapy. EV-302, a global randomized controlled trial, demonstrated that EV-PEMB, as the first-line treatment option, improved the median PFS (12.5 months vs. 6.3 months) and median OS (31.5 months vs. 16.1 months) in patients with advanced UC.

Although the clinical efficacy of EV-PEMB in treating advanced UC is superior to chemotherapy, the associated increase in healthcare costs must be considered, especially in countries with less affluent healthcare resources, such as China. Therefore, an economic evaluation of EV-PEMB is necessary to comprehensively assess its cost-effectiveness, weighing clinical advantages and potential economic implications as a first-line therapeutic option for advanced UC. The cost-effectiveness of EV-PEMB as a first-line treatment for advanced UC has not yet been evaluated. Therefore, we aim to fill this gap by evaluating the economic feasibility of EV-PEMB compared to chemotherapy in the context of the Chinese healthcare system based on the results of the EV-302 trial. Our study adheres to the Comprehensive Health Economic Evaluation and Reporting Standards (CHEERS) guidelines, ensuring transparency and methodological rigor (Husereau et al., 2022).

## 2 Methods

### 2.1 Clinical information

The clinical data used in this study were derived from the EV-302 trial (Powles et al., 2024). Specifically, the data included probabilities related to transitions in the patient health state over the course of the disease and the probability of adverse drug reactions. The enrolled population was consistent with the target group of the EV-302 trial. Inclusion criteria were adulthood, histologically unresectable or metastatic UC, and no previous systemic treatment. The main exclusion criteria included prior treatment with programmed death 1 or programmed death ligand 1 (PD-L1) inhibitors or other systemic therapies (except for neoadjuvant or adjuvant chemotherapy with recurrence occurring more than 12 months after completion of treatment), uncontrolled diabetes, persistent grade 2 or higher sensory or motor neuropathy, and a history of autoimmune diseases with prior systemic therapy within the past 2 years. Patients were randomly assigned to receive either EV-PEMB or chemotherapy, with both groups receiving treatment in 3-week cycles. Patients in the EV-PEMB group received an intravenous EV infusion (1.25 mg/kg of body weight, maximum dose 125 mg) on the first and eighth day of each cycle, and 200 mg pembrolizumab was intravenously administered after EV administration on the first day of each cycle. Patients in the chemotherapy group received gemcitabine in combination with cisplatin or carboplatin. Specifically, 1,000 mg/m^2^ body surface area gemcitabine was intravenously infused on the first and eighth day of each cycle. Cisplatin was intravenously infused at a dose of 70 mg/m^2^ body surface area on the first day of each cycle. Carboplatin was intravenously infused with an area under the curve of 5 mg/ml/min and the dosage was calculated using the Calvert formula. Treatment was continued until disease progression, development of unacceptable toxic effects, or completion of the maximum number of treatment cycles (chemotherapy, six cycles; pembrolizumab, 35 cycles; EV, no maximum). Patients included in the EV-PEMB and chemotherapy groups had similar baseline characteristics (Table 1). Based on the results of the EV-302 trial, the median duration of treatment for EV, pembrolizumab, and chemotherapy duration were 7, 8.5, and 4.1 months, respectively. The EV-302 trial did not provide comprehensive treatment data on disease progression. Therefore, all patients were presumed to receive the best supportive care (BSC) following disease progression or unacceptable toxicity occurs, which consists of palliative radiotherapy, symptom control, nutritional and psychological support (NCCN Guidelines, 2024).

**TABLE 1:** Characteristics of the patients at baseline.

Characteristic	EV-PEMB group	Chemotherapy group
Median age (year) (range)	69 (37–87)	69 (22–91)
Age ≥75	102 (23.1%)	108 (24.3%)
Sex		
Male	344 (77.8%)	336 (75.7%)
Female	98 (22.2%)	108 (24.3%)
Race or ethnic group		
Asian	99 (22.4%)	92 (20.7%)
Black	3 (0.7%)	7 (1.6%)
White	308 (69.7%)	290 (65.3%)
Other	5 (1.1%)	8 (1.8%)
Unknown or not reported	27 (6.1%)	47 (10.6%)
ECOG performance-status score		
0	223 (50.5%)	215 (48.4%)
1	204 (46.2%)	216 (48.6%)
2	15 (3.4%)	11 (2.5%)
Data missing	0	2 (0.5%)
Body-mass index		
<25	206 (46.6%)	185 (41.7%)
25 to <30	144 (32.6%)	155 (34.9%)
≥30	89 (20.1%)	101 (22.7%)
Data missing	3 (0.7%)	3 (0.7%)
Creatinine clearance		
≥60 mL/min	249 (56.3%)	257 (57.9%)
<60 mL/min	193 (43.7%)	187 (42.1%)
No. of Bajorin risk factors		
0	179 (40.5%)	183 (41.2%)
1	263 (59.5%)	259 (58.3%)
Data missing	0	2 (0.5%)
H score of nectin-4 expression		
No. of patients tested	394	406
Median score (range)	280 (0–300)	270 (0–300)
Disease status at randomization		
Locally advanced	21 (4.8%)	24 (5.4%)
Metastatic	421 (95.2%)	420 (94.6%)
Primary site of origin of disease		
Upper tract	135 (30.5%)	104 (23.4%)
Lower tract	305 (69.0%)	339 (76.4%)
Unknown	2 (0.5%)	1 (0.2%)
Histologic type		
Urothelial carcinoma	379 (85.7%)	373 (84.0%)
Urothelial carcinoma, mixed types	50 (11.3%)	53 (11.9%)
Variant urothelial carcinoma only	4 (0.9%)	7 (1.6%)
Unknown	9 (2.0%)	11 (2.5%)
Sites of metastasis		
Lymph node only	103 (23.3%)	104 (23.4%)
Visceral site	318 (71.9%)	318 (71.6%)
Bone	81 (18.3%)	102 (23.0%)
Liver	100 (22.6%)	99 (22.3%)
Lung	170 (38.5%)	157 (35.4%)
Cisplatin eligibility status		
Eligible	240 (54.3%)	242 (54.5%)
Ineligible	202 (45.7%)	202 (45.5%)
PD-L1 expression		
High, CPS ≥10	254/438 (58.0%)	254/439 (57.9%)
Low, CPS <10	184/438 (42.0%)	185/439 (42.1%)

CPS, combined positive score; ECOG, eastern cooperative oncology group; EV-PEMB, enfortumab vedotin combined with pembrolizumab; PD-L1, Programmed death ligand 1.

### 2.2 Constructing the model

We evaluated the cost-effectiveness of EV-PEMB or chemotherapy as a first-line treatment option for advanced UC using Markov models. Kaplan-Meier survival curves for PFS and OS from the EV-302 trial (Powles et al., 2024) were digitized into data points using GetData Graph Digitizer (version 1.2). Subsequently, these data points were fitted to various survival distributions using the “survival,” “survHE,” and “survminer” packages in R software, following the methodology described by Hoyle et al. (Hoyle and Henley, 2011). The best-fit survival distributions were chosen based on the Akaike information criterion (AIC) and Bayesian information criterion (BIC); lower values indicated a better fit (Williams et al., 2017). AIC and BIC values for various survival distributions of PFS and OS curves are detailed in Supplementary Table B. Ultimately, the log-logistic distribution (S(t) = (1 + (λt)^γ)) offered the best fit for the PFS and OS data (Supplementary Figure A). It was used to calculate the transition probabilities between different health states for patients during the execution of the model. The estimated shape parameter (γ) and size parameter (λ) are presented in Table 2. The model considered the background mortality rate in China (Compiled by the National Bureau of Statistics of China, 2024).

**TABLE 2:** Relevant parameters of survival distributions.

Parameters	Value	Source
Log-logistic survival model of PFS		
EV-PEMB group	Scale = 0.07657114, Shape = 1.267575	Powles et al. (2024)
Chemotherapy group	Scale = 0.1565434, Shape = 1.997612	Powles et al. (2024)
Log-logistic survival model of OS		
EV-PEMB group	Scale = 0.02895679, Shape = 1.226923	Powles et al. (2024)
Chemotherapy group	Scale = 0.06252743, Shape = 1.524174	Powles et al. (2024)

EV-PEMB, enfortumab vedotin combined with pembrolizumab; OS, overall survival; PFS, progression-free survival.

**FIGURE 1:**
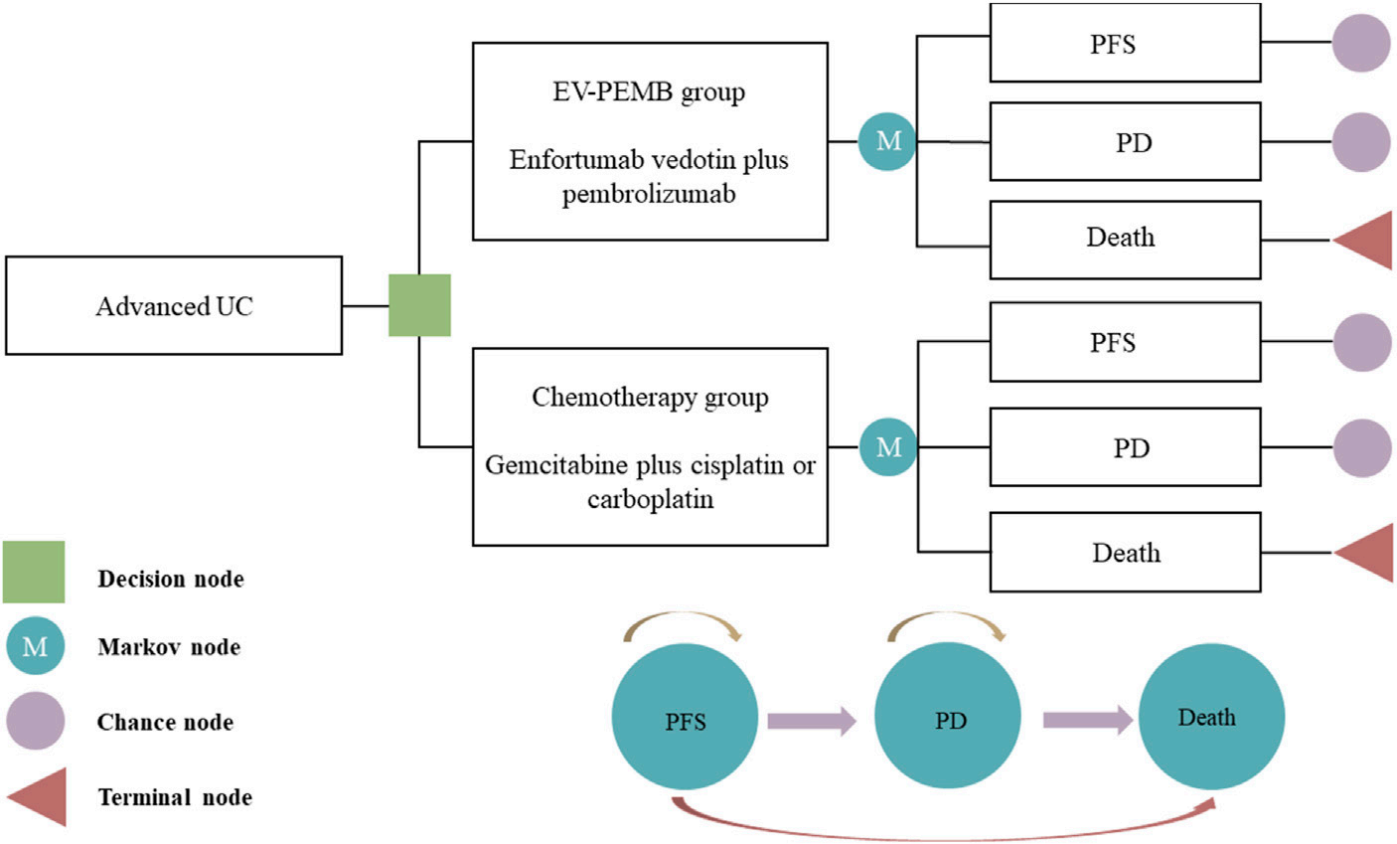
The Markov model simulating outcomes for the EV-302 trial. All patients started with PFS state and received treatment with EV-PEMB or chemotherapy. EV-PEMB, enfortumab vedotin combined with pembrolizumab; PD, progressive disease; PFS, progression-free survival; UC, urothelial carcinoma.

Our model delineated three distinct and mutually exclusive health states based on tumor progression: PFS, progressive disease (PD), and death (Figure 1). We assumed that all patients entered the model in the PFS state (Shen et al., 2022). Individuals either remained in their current health state or progressed to a different one throughout the simulation, with no option to regress to the previous state. Each cycle within the model spanned a fixed duration of 21 days. The simulation extended over 15 years, during which over 90% of patients died. In the model, the survival probabilities for each cycle for the two patient groups are detailed in Supplementary Data Sheet S2. Outcomes of the model encompassed total costs, quality-adjusted life years (QALYs), and the incremental cost-effectiveness ratios (ICERs) associated with the two treatment regimens. Following the China Pharmacoeconomic Evaluation Guidelines (Yue et al., 2021), three times the *per capita* GDP of China in 2023 was considered the willingness-to-pay (WTP) threshold ($38,133). A treatment regimen was considered cost-effective if its ICER value was below this pre-determined WTP threshold.

### 2.3 Costs and utility

This study exclusively incorporated direct medical costs, comprising drugs, tests, routine follow-up, BSC, end-of-life care, and management of grade 3 and higher adverse reactions with an incidence exceeding 5% (Table 3). Drug prices were determined from the national drug tender prices. The price of sacituzumab govitecan (an ADC approved by the FDA for UC) in China was used as the reference for EVs because EV is not yet available in China. Other data on costs were sourced from literature or expert advice and adjusted to values in 2023 based on the China Bureau of Statistics Medical Price Index (Compiled by National Bureau of Statistics of China, 2024). All costs are presented in USD, converted at the average 2023 exchange rate (1 USD = 7.03 CNY). Utility values are indicators that assess a patient’s social functioning and overall health, including physical, mental, and disease-related aspects, on a scale from 0 to 1, where 0 represents death and 1 represents optimal health. The utility values of this study assigned to PFS and PD were obtained from literature in China because of the absence of quality-of-life data in the EV-302 trial (Table 3). The detrimental utility impact of grade 3 or higher adverse reactions with an incidence exceeding 5% was considered. Costs and utilities in this study were discounted at a rate of 5% (Yue et al., 2021).

**TABLE 3:** Basic parameters of the input model and the range of sensitivity analyses.

Variable	Base value	Range		Distribution	Source
		Min	Max		
EV-PEMB group: Incidence of AEs (%)					
Maculopapular rash	7.7	6.2	9.2	Beta	Powles et al. (2024)
Anemia	3.4	2.7	4.1	Beta	Powles et al. (2024)
Hyperglycemia	5	4.0	6.0	Beta	Powles et al. (2024)
Neutropenia	4.8	3.8	5.8	Beta	Powles et al. (2024)
Neutrophil count decreased	2.5	2.0	3.0	Beta	Powles et al. (2024)
Thrombocytopenia	0.5	0.4	0.6	Beta	Powles et al. (2024)
Platelet count decreased	0	0.0	0.0	Beta	Powles et al. (2024)
Chemotherapy group: Incidence of AEs (%)					
Maculopapular rash	0	0.0	0.0	Beta	Powles et al. (2024)
Anemia	31.4	25.1	37.7	Beta	Powles et al. (2024)
Hyperglycemia	0	0.0	0.0	Beta	Powles et al. (2024)
Neutropenia	30.0	24.0	36.0	Beta	Powles et al. (2024)
Neutrophil count decreased	9.0	7.2	10.8	Beta	Powles et al. (2024)
Thrombocytopenia	19.4	15.5	23.3	Beta	Powles et al. (2024)
Platelet count decreased	6.5	5.2	7.8	Beta	Powles et al. (2024)
Cost ($)					
Gemcitabine (1000 mg)	48.1	38.5	57.7	Gamma	Yaozh (2024)
Carboplatin (100 mg)	9.2	7.4	11.0	Gamma	Yaozh (2024)
Cisplatin (10 mg)	7.1	5.7	8.5	Gamma	Yaozh (2024)
Pembrolizumab (100 mg)	2,548.8	2,039.0	3,058.6	Gamma	Yaozh (2024)
Enfortumab vedotin (30 mg)	1,593.2	1,274.6	1,911.8	Gamma	Yaozh (2024)
Maculopapular rash	73.6	58.9	88.3	Gamma	Wu et al. (2022)
Anemia	533.3	426.6	640.0	Gamma	Zheng et al. (2023)
Hyperglycemia	155.0	124.0	186.0	Gamma	Expert advice
Neutropenia	84.5	67.6	101.4	Gamma	Zheng et al. (2023)
Neutrophil count decreased	84.5	67.6	101.4	Gamma	Zheng et al. (2023)
Thrombocytopenia	1,057.2	845.8	1,268.6	Gamma	Zheng et al. (2023)
Platelet count decreased	1,057.2	845.8	1,268.6	Gamma	Zheng et al. (2023)
BSC care per cycle	182.8	146.2	219.4	Gamma	Zhang et al. (2021)
Routine follow-up per cycle	73.9	59.1	88.7	Gamma	Zhang et al. (2021)
Tests per cycle	358.4	286.7	430.1	Gamma	Liu et al. (2022b)
Terminal care in end-of-life	1,494.0	1,195.2	1,792.8	Gamma	Liu et al. (2023)
Utility value					
PFS	0.84	0.67	1.00	Beta	Xie et al. (2022)
PD	0.80	0.64	0.96	Beta	Xie et al. (2022)
Disutility due to Grade ⩾3 AEs	0.28	0.22	0.34	Beta	Wu et al. (2022)
Weight (Kg)	65.0	52.0	78.0	Normal	Liu et al. (2022a)
Creatinine clearance rate (mL/min)	70.0	56.0	84.0	Gamma	Liu et al. (2021)
Body surface area (m^2^)	1.72	1.38	2.06	Normal	Zhao et al. (2023)
Discount rate	0.05	0.00	0.08	Fixed	Yue et al. (2021)

AE, adverse event; BSC, the best supportive care; EV-PEMB, enfortumab vedotin combined with pembrolizumab; PD, progressive disease; PFS, progression-free survival.

### 2.4 Sensitivity analysis

We conducted a sensitivity analysis to assess the robustness of the results from the model. One-way sensitivity analyses were performed by adjusting parameters within specified ranges to identify those that affected the ICER. These results are represented in a tornado diagram. All parameters were varied within their 95% confidence intervals derived from the literature, using benchmark values of ±20% in the absence of data. The discount rate varied between 0% and 8% (Table 3). Probabilistic sensitivity analyses through 1,000 Monte Carlo simulations were performed to evaluate how simultaneous alterations in multiple parameters affected the model outcomes. All parameters followed pre-determined distributions, and the results from these probabilistic sensitivity analyses are presented as scatter plots (Table 3). Furthermore, the calculation of the ICER for EV-PEMB compared to chemotherapy was repeated by continuously decreasing the price of EV and pembrolizumab to determine the price of EV and pembrolizumab at which EV-PEMB could be cost-effective.

### 2.5 Subgroup analysis

Exploratory subgroup analyses were conducted to evaluate the potential effect of subgroups with varying baseline characteristics on model outcomes. Subgroups were defined based on age, sex, Eastern Cooperative Oncology Group (ECOG) performance-status score, primary site of origin of disease, PD-L1 expression, cisplatin eligibility status, site of metastasis, and renal function (Table 4). We assumed the same survival curves for all subgroups in the chemotherapy arm of the trial based on the methodology provided by Hoyle et al. (2010) as the EV-302 trial did not provide survival curves for individual subgroups and calculated ICERs and acceptable probabilities of cost-effectiveness for each subgroup using the subgroup-specific risk ratios from the EV-302 trial.

**TABLE 4:** Results of subgroup analyses.

Subgroup	PFS HR (95% CI)	OS HR (95% CI)	ICER ($/QALY)
Age			
<65	0.45 (0.32–0.62)	0.46 (0.30–0.71)	205,491.34
≥65	0.45 (0.36–0.56)	0.48 (0.38–0.63)	212,546.99
Sex			
Female	0.49 (0.34–0.71)	0.51 (0.32–0.80)	223,667.66
Male	0.44 (0.36–0.54)	0.47 (0.36–0.60)	209,145.68
ECOG performance-status score			
0	0.36 (0.28–0.48)	0.36 (0.25–0.53)	177,113.39
1 or 2	0.53 (0.42–0.68)	0.54 (0.41–0.72)	236,572.60
Primary site of origin of disease			
Upper tract	0.50 (0.35–0.71)	0.53 (0.34–0.83)	231,719.75
Lower tract	0.44 (0.35–0.54)	0.46 (0.36–0.59)	205,695.21
Liver metastases			
Present	0.53 (0.38–0.76)	0.47 (0.32–0.71)	207,112.55
Absent	0.43 (0.35–0.52)	0.47 (0.36–0.61)	209,245.32
PD-L1 expression			
Low (CPS <10)	0.50 (0.38–0.65)	0.44 (0.31–0.61)	196,413.28
High (CPS ≥10)	0.42 (0.33–0.53)	0.49 (0.37–0.66)	215,893.18
Cisplatin eligibility status			
Eligible	0.48 (0.38–0.62)	0.53 (0.39–0.72)	231,223.74
Ineligible	0.43 (0.33–0.55)	0.43 (0.31–0.59)	195,743.27
Site of metastasis			
Visceral site	0.45 (0.37–0.55)	0.47 (0.37–0.60)	209,016.85
Lymph node only	0.40 (0.26–0.62)	0.46 (0.27–0.78)	206,214.83
Renal function			
Normal	0.46 (0.30–0.71)	0.51 (0.30–0.86)	223,332.87
Mild impairment	0.46 (0.34–0.62)	0.44 (0.30–0.65)	198,087.78
Moderate or severe impairment	0.47 (0.36–0.61)	0.50 (0.37–0.69)	219,758.48

95%CI, 95% confidence interval; CPS, combined positive score; ECOG, eastern cooperative oncology group; HR, hazard ratio; ICER, incremental cost-effectiveness ratio; OS, overall survival; PD-L1, programmed death ligand 1; PFS, progression-free survival; QALY, quality-adjusted life year.

### 2.6 Scenario analysis

A scenario analysis was conducted to improve the applicability of the results of this study. In scenario 1, with no changes in treatment, model run times were adjusted to 3, 6, and 10 years to assess their effects on the model results. In scenario 2, we assumed that only 30% or 50% of patients received BSC after disease progression to model discontinuation of treatment in clinical practice by some patients due to several reasons. Scenario 3, due to unavailability of pricing for EV in China, we used prices from the US, Norway, and Japan as reference points for conducting cost-effectiveness analyses. Scenario 4, given the higher complete remission rate of EV-PEMB, we hypothesize that patients in the EV-302 trial who do not show progress by the final data collection point (8 August 2023) have achieved cure. They discontinue the aforementioned treatment but continue periodic follow-ups and undergo one examination per cycle. Adjusting patients’ survival probabilities using the background mortality rate in China, we ultimately calculate cost-effectiveness to examine potential biases in our analyses that did not account for the impact of permanent cure rates.

## 3 Results

### 3.1 Base case analysis

The results of this study are expressed in terms of total cost, QALYs, and ICER (Table 5). The EV-PEMB group achieved 3.22 QALYs at $375,420.24. In the chemotherapy group, the effectiveness was 1.70 QALYs at $23,369.67. The mean incremental effectiveness and cost in the EV-PEMB group were 1.52 QALYs and $352,050.58, respectively, relative to the chemotherapy group. The ICER for EV-PEMB *versus* chemotherapy was $232,256.16 per QALY gained. In China, EV-PEMB is unlikely to be cost-effective for treating advanced UC compared to chemotherapy when the WTP threshold is $38,133 per QALY.

**TABLE 5:** Cost and outcomes of the cost-effectiveness analyses.

Regimen	EV-PEMB	Chemotherapy	Incremental
Total QALYs	3.22	1.70	1.52
Total cost, $	375,420.24	23,369.67	352,050.58
ICER, per QALY			232,256.16

EV-PEMB, enfortumab vedotin combined with pembrolizumab; ICER, incremental cost-effectiveness ratio; QALY, quality-adjusted life year.

### 3.2 Sensitivity analysis

**FIGURE 2:**
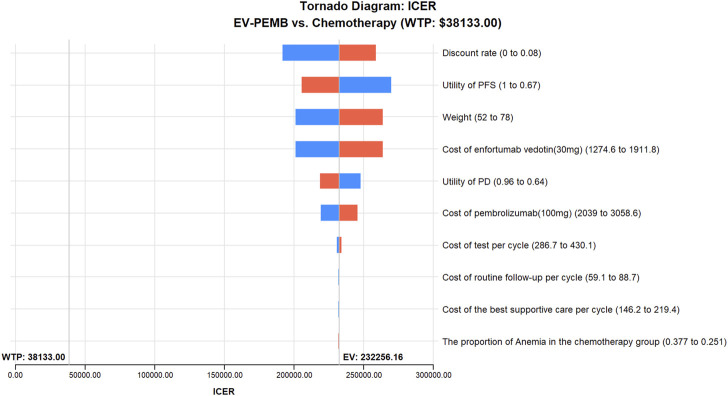
One-way sensitivity analyses of EV-PEMB in comparison to chemotherapy. EV-PEMB, enfortumab vedotin combined with pembrolizumab; ICER, incremental cost-effectiveness ratio; PD, progressive disease; PFS, progression-free survival; WTP, willingness-to-pay.

**FIGURE 3:**
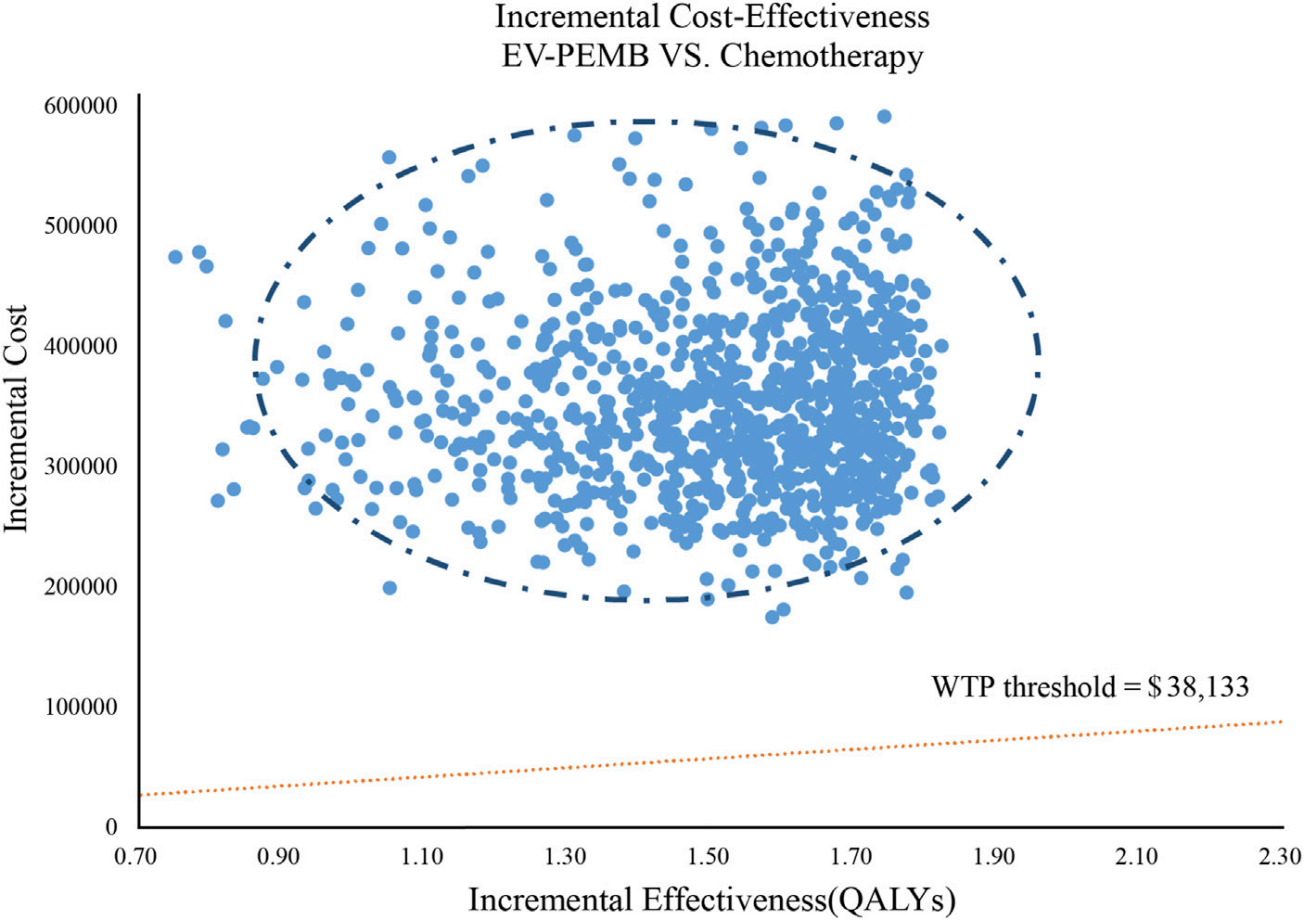
A probabilistic scatter plot of the ICER between the EV-PEMB group and the chemotherapy group. Each point means the ICER for 1 simulation. Ellipses are used to indicate 95% confidence intervals. Points that lie below the ICER threshold represent cost-effective simulations. EV-PEMB, enfortumab vedotin combined with pembrolizumab; WTP, willingness-to-pay.

The results of the one-way sensitivity analysis are shown as a tornado diagram (Figure 2). The parameters with the most significant effect on the model were the discount rate, the patient’s weight, the price of EV, and the price of pembrolizumab. When the values of these parameters were allowed to vary within a given range, the ICER was always higher than our pre-determined WTP threshold, implying that variations in the model input parameters did not affect the model results, indicating that the results are robust. The results of the probabilistic sensitivity analysis are presented in a scatter plot (Figure 3). The probability that the EV-PEMB group was cost-effective compared to the chemotherapy group was 0 at the WTP threshold of $38,133/QALY. EV-PEMB had the probability of being a cost-effective regimen for the treatment of advanced UC compared to chemotherapy only when the prices of EV and pembrolizumab simultaneously decreased to 13.1% of the original, i.e., $208.7 and $333.9 for EV and pembrolizumab, respectively.

### 3.3 Subgroup analysis

For all subgroups of the population showing different characteristics, the ICER in the EV-PEMB group was above the WTP threshold of $38,133. All subgroups had a probability of 0 for being cost-effective compared to the chemotherapy group (Table 4). The ICER was relatively low in the ECOG = 0, low PD-L1 expression, not applicable to cisplatin chemotherapy, and renal function with mild impairment populations. These results should be interpreted with caution owing to the small enrollment of the subgroups.

### 3.4 Scenario analysis

The results of the scenario analysis are shown in Table 6. In scenario 1, the ICER value of EV-PEMB gradually decreased compared to chemotherapy as the model run time increased, tending towards being more economical. In scenario 2, changes in the proportion of patients receiving BSC appeared to have little effect on the ICER value of EV-PEMB after disease progression. In Scenario 3, the EV prices were analyzed for cost-effectiveness with reference to US, Norwegian and Japanese prices, respectively, and the results show that EV-PBCM is not cost-effective. In Scenario 4, although the ICER values from the analysis are lower than the base case analysis, EV-PBCM is still not cost-effective.

**TABLE 6:** Results of scenario analyses.

Scenarios	Cost ($)		QALY		ICER ($/QALY)	Cost-effectiveness probability
	EV-PEMB	Chemotherapy	EV-PEMB	Chemotherapy		
Scenario 1						
Model runtime (year) = 3	306,337.44	17,050.50	1.67	1.21	639,367.28	0
Model runtime (year) = 6	341,561.15	20,640.90	2.40	1.49	352,037.22	0
Model runtime (year) = 10	362,894.16	22,449.49	2.90	1.63	267,258.97	0
Scenario 2						
30% of patients received BSC	361,214.43	13,548.33	3.22	1.70	229,363.62	0
50% of patients received BSC	365,273.23	16,354.42	3.22	1.70	230,190.06	0
Scenario 3						
Cost of EV = $4,334.04	783,872.31	23,369.67	3.22	1.70	501,721.73	0
Cost of EV = $1,209.46	318,233.62	23,369.67	3.22	1.70	194,528.78	0
Cost of EV = $603.95	227,997.84	23,369.66	3.22	1.70	134,998.09	0
Scenario 4	479,680.64	24,432.36	3.86	1.86	228,169.10	0

BSC, the best supportive care; EV-PEMB, enfortumab vedotin combined with pembrolizumab; ICER, incremental cost-effectiveness ratio; QALY, quality-adjusted life year.

## 4 Discussion

Chemotherapy or ICIs are the standard first-line therapeutic options for locally advanced or metastatic UC according to the guidelines for diagnosing and treating UC (2023) introduced by the Chinese Society of Clinical Oncology. However, their therapeutic efficacies are unsatisfactory (Nadal et al., 2024). ADCs (including EV) may represent a new therapeutic option. For untreated locally advanced or metastatic UC, the EV-302 trial showed that treatment with EV-PEMB was more effective than chemotherapy, with a lower incidence of grade 3 or higher adverse events than the latter (55.9% vs. 69.5%). The results of the EV-302 trial are expected to promote the use of EV-PEMB for treating advanced UC, leading to an increase in the economic burden on society and patients. This will play out as an important issue for healthcare policymakers. Therefore, an evaluation of the cost-effectiveness of EV-PEMB for treating advanced UC is necessary.

The results of our study showed that EV-PEMB cost an additional $232,256.16 per QALY compared to chemotherapy, much higher than our pre-determined WTP ($38,133/QALY). In China, EV-PEMB is not cost-effective as a first-line treatment for advanced UC, possibly due to the longer median treatment durations of EV and pembrolizumab in the EV-302 trial (7 and 8.5 months respectively) compared to chemotherapy (4.1 months). This, coupled with the fact that EV and pembrolizumab are inherently more expensive than chemotherapeutic agents, results in an EV-PEMB regimen that is far more costly to treat than chemotherapy without delivering sufficient incremental survival benefit. One-way sensitivity analyses also confirmed that the high price of EV and pembrolizumab were important influences on the lack of cost-effectiveness of EV-PEM. Therefore, addressing the high price of EV and pembrolizumab is key to making EV-PEMB cost-effective. We adjusted the prices of EV and pembrolizumab to assess cost-effectiveness. EV-PEMB was cost-effective only when the prices of both EV and pembrolizumab were reduced to 13.1% of their original prices, i.e., $208.7 and $333.9, respectively. The Chinese government has implemented an important policy since 2016, the National Drug Declaration List Negotiation, to improve drug accessibility (Zhu et al., 2022). By adopting the price negotiation policy, the state has reduced the prices of drugs, including anticancer drugs, especially innovative ones, to reduce the burden on the state, health insurance, and patients. Neither EV nor pembrolizumab has yet been included in the drug negotiation list, thereby providing great scope for the price reduction of these two drugs. Our results provide an important economic reference for the health insurance department in price negotiations.

We performed an economic evaluation of the nine subgroup populations defined in the EV-302 trial to gain insights into the cost-effectiveness of EV-PEMB in specific patient populations. Some populations, such as those with ECOG = 0, low PD-L1 expression, not applicable to cisplatin chemotherapy, and renal function with mild impairment, showed better economics than others. Physicians, patients, and policymakers may benefit from economic information for these subgroups. We also conducted a scenario analysis, which enhanced the applicability of our findings. As shown in scenario analysis 1, more than 80% of the cost-incurring years were the first 3 years. Although the cost-effectiveness probability of EV-PEMB was always 0%, which means that it was not economically superior to chemotherapy, the ICER value of EV-PEMB gradually decreased with increasing treatment time and approached the WTP value that we set. This suggests that EV-PEMB has relatively improved economics in the long term. In scenario 2, there was little change in ICER for EV-PEMB compared to chemotherapy when the proportion of patients treated with BSC after disease progression increased, suggesting that the cost outlay for BSC does not reduce the ICER of EV-PEMB. This result seems to encourage patients in the EV-PEMB group to receive possibly BSC after disease progression, consistent with scenario 1. In Scenario 3, when using prices from other countries as references for EV, the EV-PBCM remains cost-ineffective. Scenario 4’s findings indicate that not accounting for patients’ permanent cure rates does not affect the model’s outcomes, further validating the robustness of our results.

To date, only four economic evaluations of ICIs or ADCs for advanced UC from the perspective of the Chinese healthcare system have been conducted. Zhang et al. (Chen et al., 2024) and Liu S. et al. (2022) showed that atezolizumab in combination with chemotherapy as first-line treatment for metastatic UC was not cost-effective, with the price of atezolizumab having the greatest effect. Xie et al. (2022) concluded that nivolumab was not cost-effective as a maintenance treatment of advanced or metastatic UC in the total and PD-L1-positive populations at a WTP threshold of $30,447.09. Wu et al. (2022) demonstrated that EV for treating previously treated advanced UC had a 0% probability of being cost-effective. These results are consistent with our findings.

Our study has several advantages. First, this is the first assessment of the cost-effectiveness of EV-PEMB as a first-line treatment for advanced UC. This pioneering analysis is expected to guide the development of health insurance policies and clinical decisions in China and other countries. Second, the EV-302 trial directly compared EV-PEMB to chemotherapy, and our study used the most recent survival data available from the EV-302 trial for a cost-effectiveness analysis. Third, 21.6% of the participants in the EV-302 trial were from Asia, so the findings largely apply to the Chinese context. Undeniably, there are some limitations in our study, first, due to the lack of survival data after the follow-up period, our long-term survival data were simulated by survival modeling, which may introduce some bias in the model results. We plan to refine the cost-effectiveness analysis when long-term survival data are available. Second, due to the lack of detailed treatment data after patients’ disease progression, we assumed that all patients received BSC as a second-line treatment regimen, which may be biased from clinical practice. However, such an assumption would not change the model results when the one-way sensitivity results suggest otherwise. Third, our analysis only considered grade ≥3 adverse reactions with an incidence of more than 5%. However, sensitivity analyses confirmed that changes in the probability of occurrence of these adverse reactions would not affect the results.

## 5 Conclusion

In summary, from the perspective of the Chinese healthcare system, EV-PEMB is not cost-effective as a first-line treatment strategy for advanced UC compared to chemotherapy. Substantial reductions in the price of EV and pembrolizumab are necessary to make EV-PEMB cost-effective. These findings provide essential economic evidence for healthcare providers and patients to assess the suitability of this treatment option. Furthermore, our research supports the healthcare insurance sector in China with crucial economic analysis for pricing strategies following the introduction of EV. From a behavioral standpoint, these results may influence healthcare providers’ and patients’ attitudes towards treatment choices, encouraging greater consideration of cost-effectiveness and long-term outcomes.

## Data Availability

The original contributions presented in the study are included in the article/Supplementary Material, further inquiries can be directed to the corresponding author.
